# Association between dietary fiber intake and atherosclerotic cardiovascular disease risk in adults: a cross-sectional study of 14,947 population based on the National Health and Nutrition Examination Surveys

**DOI:** 10.1186/s12889-022-13419-y

**Published:** 2022-05-31

**Authors:** Shutang Zhang, Jie Tian, Min Lei, Canye Zhong, Yan Zhang

**Affiliations:** 1 Department of Geriatrics, Chongqing University Fuling Hospital, Chongqing Clinical Research Center for Geriatric Diseases, Chongqing, 408000 People’s Republic of China; 2 Department of Cardiovascular Medicine CCU, Hanzhong People’s Hospital, No.251 North Unity Street, Hantai District, Hanzhong, 723000 Shaanxi People’s Republic of China

**Keywords:** Dietary fiber intake, Framingham risk score, Cardiovascular disease, 10-year risk

## Abstract

**Background:**

This study aimed to investigate the association between dietary fiber intake and long-term cardiovascular disease (CVD) risk based on the National Health and Nutrition Examination Survey (NHANES) database.

**Methods:**

A total of 14,947 participants aged 20–79 from the NHANES database were included in this study between 2009 and 2018. The atherosclerotic cardiovascular disease (ASCVD) score was utilized to predict the 10-year risk of CVD in individuals (low, borderline, intermediate, and high risk). Weighted univariate and multinomial multivariate logistic regression analyses were used to analyze the association between dietary fiber intake and long-term CVD risk.

**Results:**

Higher dietary fiber density may be associated with a reduced ASCVD risk in participants with intermediate risk [odds ratio (OR) = 0.76; 95% confidence interval (CI), 0.61–0.94] and high risk (OR = 0.60; 95%CI, 0.45–0.81) compared with those in the group with low risk. Higher total dietary fiber intake may also reduce ASCVD risk in participants with high risk (OR = 0.84; 95%CI, 0.75–0.95). Subgroup analyses showed that higher dietary fiber density may be related to reduced ASCVD risk in intermediate-risk participants aged 20–39 (OR = 0.62; 95%CI, 0.43–0.89) and 40–59 (OR = 0.67; 95%CI, 0.49–0.94). In high-risk participants, higher dietary fiber density may reduce ASCVD risk in 20–39-year-old (OR = 0.38; 95%CI, 0.19–0.77), 40–59-year-old (OR = 0.37; 95%CI, 0.20–0.70), male (OR = 0.47; 95%CI, 0.23–0.97) and female (OR = 0.57; 95%CI, 0.38–0.86) participants.

**Conclusion:**

Higher dietary fiber density and total dietary fiber intake were associated with a lower long-term CVD risk, especially in the 20–39 and 40–59 age groups, where the reduction was most significant.

**Supplementary Information:**

The online version contains supplementary material available at 10.1186/s12889-022-13419-y.

## Introduction

Cardiovascular diseases (CVD), the world’s leading cause of death, are a group of disorders of the heart and blood vessels, including coronary heart disease, cerebrovascular disease, rheumatic heart disease and other diseases, claiming an estimated 17.9 million lives each year [1, 2]. CVD presents a heavy burden for the world due to its high treatment cost and extensive preventive interventions [3, 4]. Evidence demonstrated that the occurrence of most CVD can be attributed to a series of factors, such as smoking, obesity, diabetes, dyslipidemia, hypertension, diet, excessive alcohol consumption, and mental state [5, 6]. Early prevention can effectively reduce the incidence of CVD, but CVD-related deaths still account for a large proportion of all-cause deaths.

Dietary fiber can affect the cardiometabolic pathways, improve lipid or lipoprotein metabolism, insulin homeostasis, and so on [7]. Epidemiologic studies have shown that dietary fiber intake is associated with the CVD risk in short and medium-term follow-up [8–10]. Murai et al. indicated that seaweed intake was inversely associated with the risk of ischemic heart disease [8]. Song et al. found that total fruit and whole fruit intake were inversely related to cardiovascular risk factors such as obesity, metabolic syndrome and hypertension [9]. Wang et al. showed that higher fiber intake and fiber intake density may be associated with a lower risk of major adverse cardiovascular events [10]. An in-depth understanding of the role of dietary fiber intake in predicting the long-term CVD risk can help the public identify optimal dietary patterns and improve long-term survival. The atherosclerotic cardiovascular disease (ASCVD) score, recommended by the American College of Cardiology (ACC) and American Heart Association (AHA), is a commonly and widely used to evaluate the 10-year CVD [11]. In this study, we applied this score to identify people at high risk of CVD over the next ten years and assessed the association between dietary fiber intake and the CVD risk based on the National Health and Nutrition Examination Survey (NHANES) database.

## Methods

### Study population

Data in this study were extracted from the NHANES database between 2009 and 2018, which is a cross-sectional survey of the health and nutrition status of the U.S. civilian and non-institutionalized population conducted by the National Center of Health Statistics (NCHS) and the Centers for Disease Control and Prevention (CDC). Subjects were randomly screened based on a complex, stratified multi-stage cluster sampling design. The information collection was carried out through interviews. Additional information was available at: https://www.cdc.gov/nchs/tutorials/dietary/SurveyOrientation/Resource Dietary Analysis/intro.htm. A total of 14,947 participants with complete data were included in this study.

### Data collection

Participants’ information including age (20–79 years old), gender (male and female), body mass index (BMI, kg/m^2^), race (Mexican Americans, Hispanics, non-Hispanic whites, non-Hispanic blacks, and others), marital status (married, widowed, divorced/separated, and unmarried), education level (< high school, high school/GED, and > high school), family income (< 20,000$ and ≥ 20,000$), smoking status (yes and no), hypertension (yes and no), diabetes (yes and no), metabolic syndrome (yes and no), use of high blood pressure medication (yes and no), now increasing exercise (yes and no), systolic blood pressure (SBP), diastolic blood pressure (DBP), high-density lipoprotein (HDL), total cholesterol (TC), total bilirubin, creatinine (Cr), total energy, total dietary fiber intake, and dietary fiber density was collected.

### Definition

The data on dietary fiber intake were obtained through two 24-h dietary recall interviews. The first dietary recall interview was conducted in the mobile examination center (MEC), and the second interview was conducted using phones 3 to 10 days later. The first dietary recall interview was a face-to-face interview. A set of measurement guidelines (various glasses, bowls, mugs, bottles, household spoons, measuring cups and spoons, a ruler, thickness sticks, bean bags, and circles) was available in the MEC dietary interview room for participants to use to report the amount of food. There were more checks on weekends than on weekdays, and food intake may vary between weekdays and weekends. Therefore, the use of the MEC weight disproportionately represents weekend intake. Dietary fiber intake was calculated according to the United States Department of Agriculture (USDA) food and nutrient database for dietary studies [1]. Total dietary fiber intake was obtained based on an average of the two interviews. Dietary fiber density (10 g/1000 kcal) was defined as the ratio of dietary fiber intake to total energy intake.

Smoking status was confirmed according to two items, including SMQ020 (Have you smoked at least 100 cigarettes in your lifetime?) and SMQ935 (Do you smoke cigarettes now?). The subjects were divided into a smoking group (meeting the two items) and a non-smoking group (meeting items ≤ 1).

### ASCVD score

The ASCVD risk score was utilized to predict the 10-year risk of CVD in individuals based on the age, sex, race, cholesterol levels, blood pressure, medication use, diabetic status, and smoking status of the participants [11]. The predictive criteria of the 10-year risk of CVD were as follows: (1) low risk (< 5%); (2) borderline risk (5% to 7.5%); (3) intermediate risk (≥ 7.5% to < 20%); (4) high risk (≥ 20%). The participants with low risk served as the control group for CVD, and others with borderline/intermediate/high risk served as the case group.

### Statistical analysis

Shapiro–Wilk test was conducted to test the normality of the data. Measurement data with normal distribution were described by mean ± standard deviation (SD). The t-test was used for comparison between the two groups, and analysis of variance was used for comparison between multiple groups. Data with abnormal distribution were presented by the median and interquartile range [M (Q1, Q3)]. The Man-Whitney U rank-sum test was used for comparison between two groups, and the Kruskal–Wallis H rank-sum test was used for comparison between multiple groups. Enumeration data were described by the numbers and percentage [n (%)]. Chi-square test or Fisher’s exact probability test was used to perform the comparison between groups. All statistical analyses were performed by SAS9.4 software (SAS Institute Inc., Cary, NC, USA) using a two-sided test. *P*-value < 0.05 was considered statistically significant.

Differences between the low-risk, borderline-risk, intermediate-risk, and high-risk groups were analyzed to find possible confounders. The association between dietary fiber density and total dietary fiber and long-term CVD risk was analyzed in different CVD risk groups. Model 1 was a weighted univariate multinomial logistic regression model. Model 2 was a weighted multinomial multivariate logistic regression model that adjusted for age, gender, family income, education levels, and marital status. Model 3 was a weighted multinomial multivariate logistic regression model that adjusted for age, gender, family income, education levels, marital status, total bilirubin, creatinine, and metabolic syndrome. The normality test for continuous variables was shown in Supplement Fig. 1. The multicollinearity diagnosis for weighted models was presented in Supplement Table 1.

## Results

### Baseline characteristics of participants

**Fig. 1:**
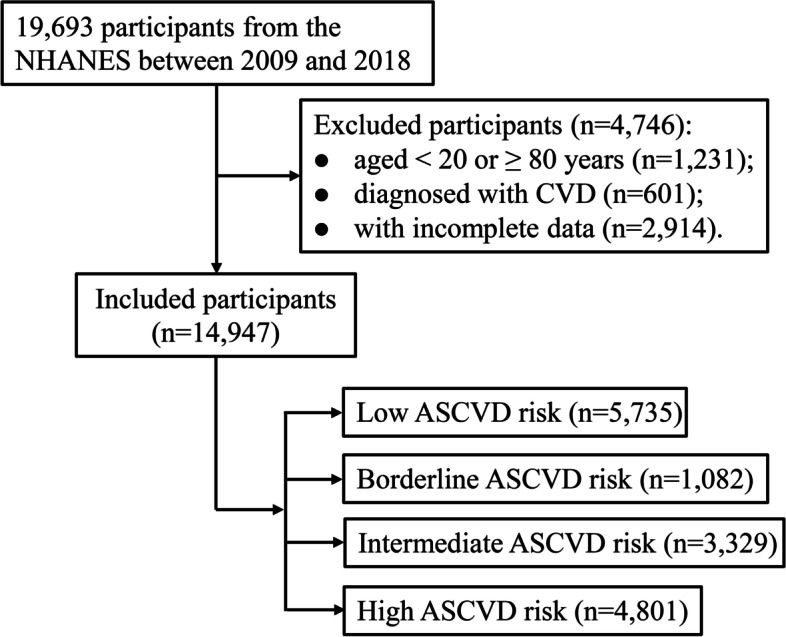
Flow chart of the study population. ASCVD, atherosclerotic cardiovascular disease; NHANES, National Health and Nutrition Examination Survey

A total of 19,693 participants were extracted from the NHANES database, 1,231 participants aged < 20 or ≥ 80, 601 participants diagnosed with CVD, and 2,914 participants with incomplete data were excluded. Finally, 14,947 participants were included in the study (Fig. 1). Among the included participants, the median age was 46.00 (33.00, 60.00) years, including 7,183 (48.06%) males and 7,764 (51.94%) females. The median total dietary fiber intake and dietary fiber density were 15.45 (10.75, 21.85) g and 0.78 (0.58, 1.06) 10 g/1000 kcal, respectively. According to the ASCVD, the predicted number of participants at low risk, borderline risk, intermediate risk, and high risk for CVD over the next 10 years were 5,735 (38.37%), 1,082 (7.24%), 3,329 (22.27%), and 4,801 (32,12%), respectively. The characteristics of individuals were shown in Table 1.

**Table 1:** Difference analysis between different atherosclerotic cardiovascular disease (ASCVD) risk groups

Characteristics	Total (*n* = 14,947)	Low risk group (*n* = 5735)	Borderline risk group (*n* = 1082)	Intermediate risk group (*n* = 3329)	High risk group (*n* = 4801)	Statistics	*P*
Age, M (Q_1_, Q_3_)	46.00 (33.00, 60.00)	32.00 (25.00, 40.00)	43.00 (34.00, 50.00)	50.00 (41.00, 57.00)	64.00 (57.00, 70.00)	χ^2^ = 8719.967	< 0.001
Gender, n (%)						χ^2^ = 533.159	< 0.001
Female	7764 (51.94)	3468 (60.47)	622 (57.49)	1821 (54.70)	1853 (38.60)		
Male	7183 (48.06)	2267 (39.53)	460 (42.51)	1508 (45.30)	2948 (61.40)		
BMI, kg/m^2^, mean ± SD	29.37 ± 6.97	28.23 ± 7.23	30.24 ± 7.41	30.56 ± 7.15	29.70 ± 6.19	F = 94.140	< 0.001
Race, n (%)						χ^2^ = 264.033	< 0.001
Mexican Americans	2242 (15.00)	936 (16.32)	178 (16.45)	508 (15.26)	620 (12.91)		
Hispanics	1531 (10.24)	480 (8.37)	101 (9.33)	356 (10.69)	594 (12.37)		
Non-Hispanic whites	6183 (41.37)	2107 (36.74)	469 (43.35)	1416 (42.54)	2191 (45.64)		
Non-Hispanic blacks	3140 (21.01)	1290 (22.49)	202 (18.67)	643 (19.32)	1005 (20.93)		
Others	1851 (12.38)	922 (16.08)	132 (12.20)	406 (12.20)	391 (8.14)		
Marital status, n (%)						χ^2^ = 1902.546	< 0.001
Married	7776 (52.02)	2548 (44.43)	581 (53.70)	1844 (55.39)	2803 (58.38)		
Widowed	689 (4.61)	32 (0.56)	22 (2.03)	135 (4.06)	500 (10.41)		
Divorced/separation	2114 (14.14)	507 (8.84)	157 (14.51)	589 (17.69)	861 (17.93)		
Unmarried	4368 (29.22)	2648 (46.17)	322 (29.76)	761 (22.86)	637 (13.27)		
Education level, n (%)						χ^2^ = 331.585	< 0.001
< high school	2899 (19.40)	806 (14.05)	203 (18.76)	727 (21.84)	1163 (24.22)		
High school/GED	3323 (22.23)	1085 (18.92)	262 (24.21)	769 (23.10)	1207 (25.14)		
> high school	8725 (58.37)	3844 (67.03)	617 (57.02)	1833 (55.06)	2431 (50.64)		
Income family, n (%)						χ^2^ = 64.207	< 0.001
< 20,000 $	12,205 (81.66)	4844 (84.46)	892 (82.44)	2703 (81.20)	3766 (78.44)		
≥ 20,000 $	2742 (18.34)	891 (15.54)	190 (17.56)	626 (18.80)	1035 (21.56)		
Smoking, n (%)						χ^2^ = 6.324	0.097
Yes	10,493 (70.20)	3980 (69.40)	757 (69.96)	2321 (69.72)	3435 (71.55)		
No	4454 (29.80)	1755 (30.60)	325 (30.04)	1008 (30.28)	1366 (28.45)		
Hypertension, n (%)						χ^2^ = 1857.847	< 0.001
Yes	4669 (31.24)	725 (12.64)	278 (25.69)	1216 (36.53)	2450 (51.03)		
No	10,278 (68.76)	5010 (87.36)	804 (74.31)	2113 (63.47)	2351 (48.97)		
Diabetes, n (%)						χ^2^ = 335.823	< 0.001
Yes	1695 (11.34)	310 (5.41)	132 (12.20)	510 (15.32)	743 (15.48)		
No	13,252 (88.66)	5425 (94.59)	950 (87.80)	2819 (84.68)	4058 (84.52)		
Metabolic syndrome, n (%)						χ^2^ = 916.488	< 0.001
Yes	12,957 (86.69)	5487 (95.68)	979 (90.48)	2855 (85.76)	3636 (75.73)		
No	1990 (13.31)	248 (4.32)	103 (9.52)	474 (14.24)	1165 (24.27)		
Use of high blood pressure medication, n (%)						χ^2^ = 575.659	< 0.001
Yes	905 (6.05)	65 (1.13)	47 (4.34)	204 (6.13)	589 (12.27)		
No	14,042 (93.95)	5670 (98.87)	1035 (95.66)	3125 (93.87)	4212 (87.73)		
Now increasing exercise, n (%)						χ^2^ = 1.019	0.797
Yes	567 (79.52)	87 (78.38)	40 (85.11)	159 (79.50)	281 (79.15)		
No	146 (20.48)	24 (21.62)	7 (14.89)	41 (20.50)	74 (20.85)		
SBP, mmHg, mean ± SD	122.67 ± 17.23	114.15 ± 12.14	119.23 ± 13.30	122.92 ± 15.16	133.44 ± 18.54	F = 1424.593	< 0.001
DBP, mmHg, mean ± SD	71.10 ± 12.15	68.72 ± 10.92	72.64 ± 11.25	73.12 ± 11.56	72.20 ± 13.59	F = 126.362	< 0.001
HDL mg/dl, mean ± SD	53.26 ± 16.12	56.07 ± 15.58	52.77 ± 16.01	51.15 ± 15.29	51.49 ± 16.85	F = 98.361	< 0.001
TC, mmol/l, mean ± SD	193.53 ± 41.65	179.56 ± 35.02	194.59 ± 35.97	201.79 ± 40.34	204.24 ± 46.07	F = 393.357	< 0.001
Total bilirubin, umol/L, M (Q_1_, Q_3_)	10.26 (6.84, 13.68)	10.26 (6.84, 13.68)	10.26 (6.84, 13.68)	10.26 (6.84, 11.97)	10.26 (8.55, 13.68)	χ^2^ = 32.228	< 0.001
Cr, mg/dL, M (Q_1_, Q_3_)	0.84 (0.71, 0.99)	0.79 (0.67, 0.93)	0.82 (0.69, 0.96)	0.83 (0.71, 0.97)	0.90 (0.77, 1.07)	χ^2^ = 826.562	< 0.001
Total energy, kcal, M (Q_1_, Q_3_)	1956.00 (1504.00, 2517.50)	1983.00 (1532.00, 2549.50)	1946.25 (1526.00, 2524.00)	1975.00 (1516.50, 2534.00)	1911.50 (1458.50, 2469.50)	χ^2^ = 31.348	< 0.001
Total dietary fiber intake, g, M (Q_1_, Q_3_)	15.45 (10.75, 21.85)	15.40 (10.85, 21.60)	15.10 (10.70, 22.20)	15.30 (10.60, 21.90)	15.65 (10.75, 21.95)	χ^2^ = 1.106	0.776
Dietary fiber density, 10 g/1000 kcal, M (Q_1_, Q_3_)	0.78 (0.58, 1.06)	0.78 (0.57, 1.03)	0.76 (0.58, 1,04)	0.77 (0.57, 1.05)	0.82 (0.60, 1.10)	χ^2^ = 36.806	< 0.001

*BMI* Body mass index, *SBP* Systolic blood pressure, *DBP* Diastolic blood pressure, *HDL* High-density lipoprotein, *TC* Total cholesterol, *Cr* Creatinine

Difference analysis between the low-risk, borderline-risk, intermediate-risk, and high-risk ASCVD groups showed statistical difference in age, gender, BMI, race, marital status, education level, family income, hypertension, diabetes, metabolic syndrome, use of high blood pressure medication, SBP, DBP, HDL, TC, total bilirubin, creatinine, total energy, and dietary fiber density among the four groups (all *P* < 0.001). However, no statistical difference was found in total dietary fiber intake among the four groups (*P* = 0.776; Table1).

### Association of dietary fiber density and total dietary fiber with ASCVD risk

**Fig. 2:**
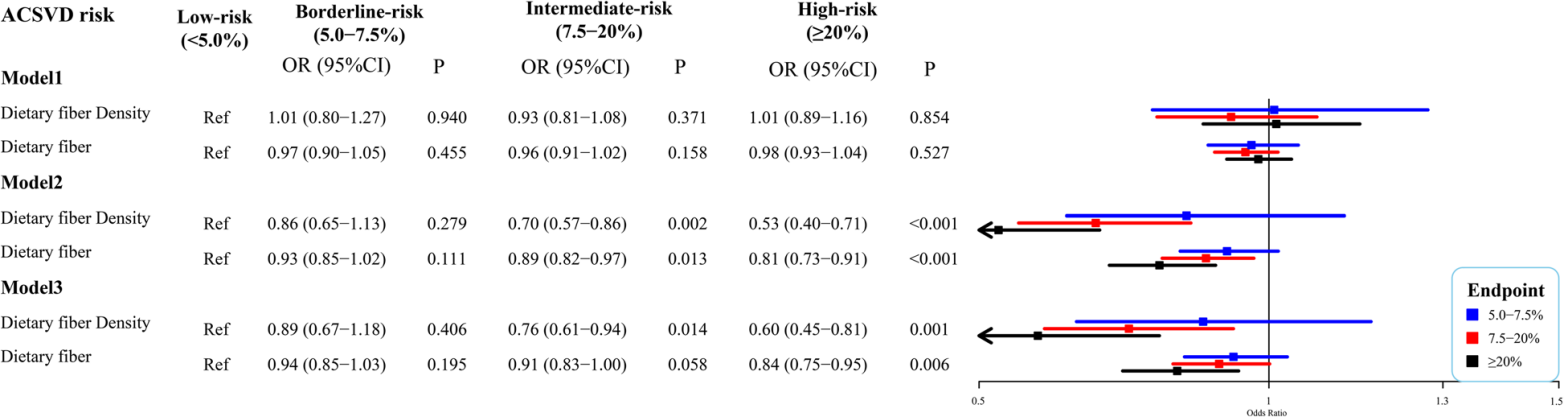
Weighted logistic regression analysis between dietary fiber and cardiovascular disease (CVD) risk. Model 1, weighted univariate multinomial logistic regression model; Model 2, weighted multinomial multivariate logistic regression model that adjusted for age, gender, family income, education levels, and marital status; Model 3, weighted multinomial multivariate logistic regression model that adjusted for age, gender, family income, education levels, marital status, total bilirubin, creatinine, and metabolic syndrome

The relationships between dietary fiber density and ASCVD risk were shown in Fig. 2. There were no statistically significant between dietary fiber density and ASCVD risk in different risk groups (model 1; *P* > 0.05). After adjustment for age, gender, family income, education levels, and marital status (model 2), higher dietary fiber density may reduce the ASCVD risk in participants with intermediate risk [odds ratio (OR) = 0.70; 95% confidence interval (CI), 0.57–0.86] and high risk (OR = 0.53; 95%CI, 0.40–0.71) compared with those in low-risk group. After further adjustment for total bilirubin, creatinine, and metabolic syndrome (model 3), higher dietary fiber density was still associated with a reduced ASCVD risk in participants with intermediate-risk (OR = 0.76; 95%CI, 0.61–0.94) and high-risk (OR = 0.60; 95%CI, 0.45–0.81).

The association between total dietary fiber intake and ASCVD risk was also analyzed (Fig. 2). Compared with participants in the ASCVD low-risk group, higher total dietary fiber intake was related to a reduced ASCVD risk in participants with intermediate risk (OR = 0.89; 95%CI, 0.82–0.97) and high risk (OR = 0.81; 95%CI, 0.73–0.91) when adjustment for age, gender, family income, education levels, and marital status. After further adjustment for total bilirubin, creatinine, and metabolic syndrome, higher total dietary fiber intake may still reduce ASCVD risk in participants with high risk (OR = 0.84; 95%CI, 0.75–0.95), while no statistical significance was found among participants in the intermediate-risk group (*P* = 0.058).

### Further analysis of the relationship between dietary fiber density and ASCVD risk based on age and gender

**Fig. 3:**
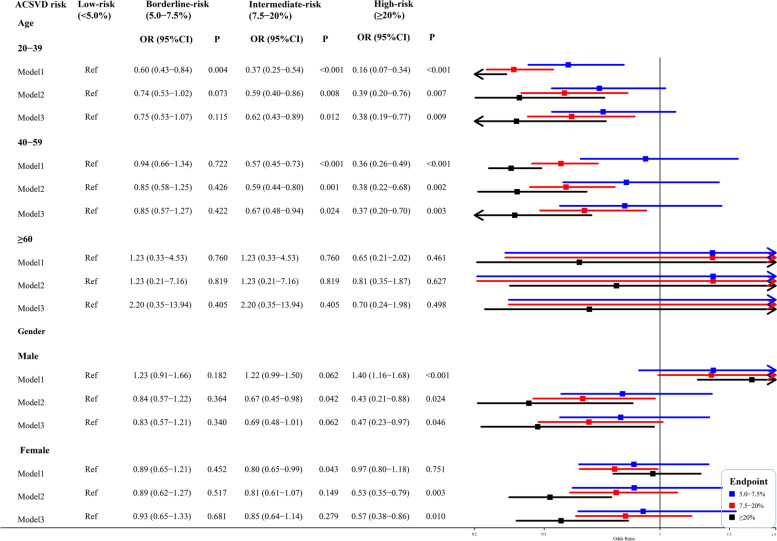
Weighted logistic regression analysis between dietary fiber density and CVD risk in age and gender subgroups. Model 1, weighted univariate multinomial logistic regression model; Model 2, weighted multinomial multivariate logistic regression model that adjusted for age/gender, family income, education levels, and marital status; Model 3, weighted multinomial multivariate logistic regression model that adjusted for age/gender, family income, education levels, marital status, total bilirubin, creatinine, and metabolic syndrome

As summarized in Fig. 3, subgroup analysis was to further explore the relationship between dietary fiber density and ASCVD risk in age and gender subgroups. The results showed that higher dietary fiber density was associated with a reduced ASCVD risk in intermediate-risk participants aged 20–39 (OR = 0.62; 95%CI, 0.43–0.89) and 40–59 (OR = 0.67; 95%CI, 0.49–0.94) after adjustment for all confounders, while no statistical significances were observed in participants aged ≥ 60 (*P* = 0.405), males (*P* = 0.062) and females (*P* = 0.279). Compared with participants in the low-risk group, higher dietary fiber density may also reduce ASCVD risk in high-risk 20–39-year-old (OR = 0.38; 95%CI, 0.19–0.77), 40–59-year-old (OR = 0.37; 95%CI, 0.20–0.70), male (OR = 0.47; 95%CI, 0.23–0.97) and female (OR = 0.57; 95%CI, 0.38–0.86) participants after adjustment for all confounders, while no statistical significance was found in participants aged ≥ 60 (*P* = 0.498).

## Discussion

In this study, we analyzed the association between dietary fiber density and total dietary fiber and long-term CVD risk in different ASCVD risk groups based on a large sample from the NHANES database. Our results found that both higher dietary fiber density and total dietary fiber were associated with a reduced long-term ASCVD risk in the intermediate-risk and high-risk groups. Subgroup analyses showed that higher dietary fiber density was still related to a reduced ASCVD risk in intermediate-risk and high-risk participants aged 20–39 and 40–59, as well as in high-risk male and female participants.

Dietary fiber has been shown to have multiple health benefits, but the average daily intake for most Americans is 15 g/day, which is below the recommended amount [12]. According to the results of epidemiological studies on the protective effect of dietary fiber intake, the recommended dietary reference intake of dietary fiber is 14 g/1000 kcal [13]. Our results showed that higher dietary fiber density and total dietary fiber intake were associated with a lower long-term CVD risk. Previous studies have focused on the association between dietary fiber intake and short-term and medium-term CVD risk [8–10], while our results provided the relationship between dietary fiber density and total dietary fiber intake and long-term CVD risk. Numerous studies suggested that total dietary fiber was inversely related to the risk of weight gain [14], coronary heart disease [15], high blood pressure [16], and CVD death [17]. Several biological mechanisms may explain the association between higher dietary fiber intake and lower CVD risk. First, dietary fiber may reduce the CVD risk by reducing the coagulation activity of type 1 plasminogen activator inhibitor and coagulation factor VII [18, 19]. Second, higher dietary fiber intake may be related to lower inflammatory response. Several studies have reported that higher dietary fiber intake can reduce the levels of inflammatory markers such as C-reactive protein [20, 21]. Third, the protective effect of dietary fiber on CVD may be associated with metabolic diseases, that is, dietary fiber may regulate the intestinal microbiota, which plays an important role in the development of metabolic diseases such as atherosclerosis, obesity and type 2 diabetes [22–24].

Our results found that higher dietary fiber density was significantly associated with a lower CVD risk in participants aged 20–39 and 40–59. The possible explanation was that the relationship between high dietary fiber intake and low CVD risk was related to the general health of the population, the absorption of fiber, and the incidence of obesity. Edwards et al. demonstrated that young people in many countries had insufficient intake of dietary fiber [25]. Yamada et al. indicated that adults aged 30–40 had a rapid increase in BMI [26]. These may be due to the fact that the consumption of a large number of refined carbohydrates, lipids, and low dietary fiber foods was conducive to weight gain [27–29]. In addition, dietary fiber has been used for the prevention and treatment of obesity [30, 31]. Studies have shown that obesity is an important risk factor for CVD [32, 33]. The type and absorption of dietary fiber may also affect the CVD risk. McKeown et al. demonstrated that cereal fiber intake was associated with a reduction in the prevalence of metabolic syndrome, but not with total fiber and fruit fiber intake [34]. Mirmiran et al. found that the intake of different types of dietary fiber was related to a reduced CVD risk, especially soluble dietary fiber [35]. Our results may also indicate that the earlier intake of high dietary fiber, the better the protection against CVD.

Some strengths were presented in this study, we analyzed the impact of dietary fiber density and total dietary fiber on long-term CVD risk in different risk groups based on ASCVD. Dietary fiber density considered the factor of energy, which can better reflect the overall situation of individual dietary fiber in daily diet. Therefore, further analysis was performed to explore the relationship between dietary fiber density and ASCVD risk in age and gender subgroups. However, this study had some limitations. First, the effect of insoluble and soluble fiber intake on CVD risk could not be analyzed because of the lack of data. Second, we did not analyze the effect of different dietary fiber intake doses on CVD risk. Third, a dietary fiber intake of 14 g/1000 kcal had a better protective effect [12, 13], while the median dietary fiber intake of our study population was 7.81 g/1000 kcal, which may reduce the accuracy of our results. Fourth, some variables related to CVD, such as genetic factors could not be analyzed due to the database limitations. Fifth, mental state and sleep duration were associated with CVD risk [36, 37], but we did not analyze the effects of these variables, which may be potentially confounding.

## Conclusion

Higher dietary fiber density and total dietary fiber were associated with a lower long-term CVD risk. Higher dietary fiber density was most significantly related to a lower ASCVD risk in people aged 20–39 and 40–59. Young people may benefit more from a high intake of dietary fiber to protect against CVD.

## Supplementary Information

**Additional file 1.**

**Additional file 2: Supplement Table 1.** Multicollinearity diagnosis for weighted models.

## Data Availability

The datasets generated and/or analyzed during the current study are available in the National Health and Nutrition Examination Survey public database, https://www.cdc.gov/nchs/nhanes/index.htm.

## Abbreviations

CVD: Cardiovascular diseases
NHANES: National Health and Nutrition Examination Survey
NCHS: National Center of Health Statistics
CDC: Centers for Disease Control and Prevention
MEC: Mobile examination center
USDA: United States Department of Agriculture
TC: Total cholesterol
HDL: High-density lipoprotein
FRS: Framingham risk score
SD: Standard deviation
M (Q1, Q3): Median and interquartile range
