# Supplementary figures and images for: Association between dietary fiber intake and atherosclerotic cardiovascular disease risk in adults: a cross-sectional study of 14,947 population based on the National Health and Nutrition Examination Surveys
Source: BMC Public Health. 2022 May 31;22:1076. doi: 10.1186/s12889-022-13419-y (PMC9158217; doi:10.1186/s12889-022-13419-y)

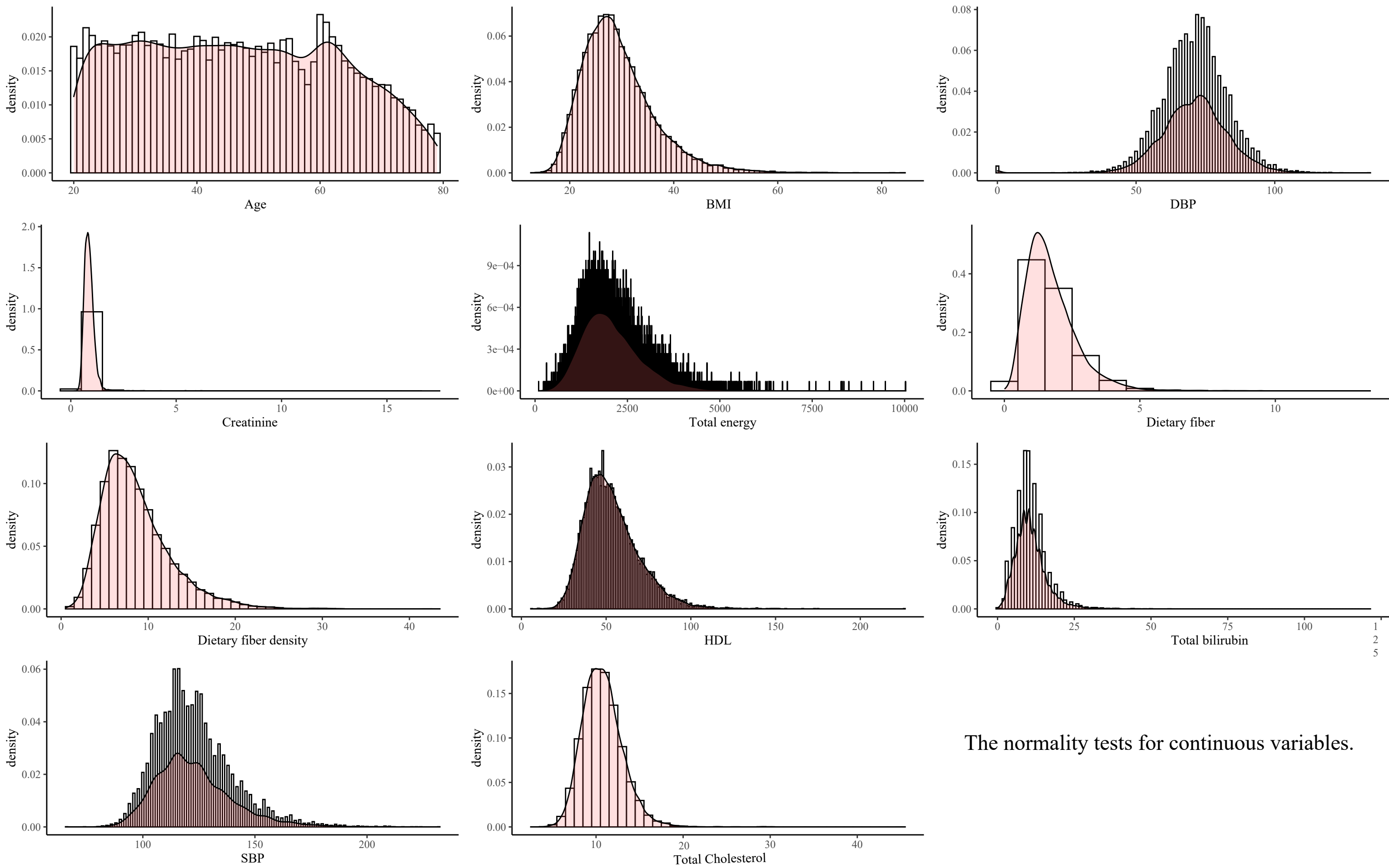

The normality tests for continuous variables.

Supplement: Supplementary file 1 — Additional file 1. [file 12889_2022_13419_MOESM1_ESM.pdf]
