# Supplementary material for: Association between dietary fiber intake and atherosclerotic cardiovascular disease risk in adults: a cross-sectional study of 14,947 population based on the National Health and Nutrition Examination Surveys
Source: BMC Public Health. 2022 May 31;22:1076. doi: 10.1186/s12889-022-13419-y (PMC9158217; doi:10.1186/s12889-022-13419-y)
Supplement: Supplementary file 2 — Additional file 2: Supplement Table 1. Multicollinearity diagnosis for weighted models. [file 12889_2022_13419_MOESM2_ESM.docx]

**Supplement Table 1.** Multicollinearity diagnosis for weighted models

| Variables | GVIF | Df | GVIF^(1/(2*Df)) |
| --- | --- | --- | --- |
| **Dietary fiber density** | 1.80 | 1 | 1.34 |
| Age | 4.05 | 1 | 2.01 |
| Gender | 3.45 | 1 | 1.86 |
| Family income | 1.43 | 1 | 1.2 |
| Education levels | 2.12 | 2 | 1.21 |
| creatinine | 1.94 | 1 | 1.39 |
| Marital status | 3.57 | 3 | 1.24 |
| Metabolic syndrome | 1.36 | 1 | 1.16 |
| Total bilirubin | 1.35 | 1 | 1.16 |
|  |  |  |  |
| **Total dietary fiber** | 2.17 | 1 | 1.47 |
| Age | 1.63 | 1 | 1.27 |
| Gender | 4.38 | 1 | 2.09 |
| Family income | 3.90 | 1 | 1.98 |
| Education levels | 1.44 | 1 | 1.20 |
| Creatinine | 2.07 | 2 | 1.20 |
| Marital status | 1.33 | 1 | 1.15 |
| Metabolic syndrome | 3.42 | 3 | 1.23 |
| Total bilirubin | 1.36 | 1 | 1.17 |
